# Vitamin D supplementation is beneficial in improving the prognosis of patients with acute respiratory failure in the intensive care unit: a retrospective study based on the MIMIC-IV database

**DOI:** 10.3389/fmed.2023.1271060

**Published:** 2023-11-23

**Authors:** Song Hu, Qian He, Jun Xie, Hui Liu, Rong Zhou, Chong Li

**Affiliations:** Department of Respiratory and Critical Care Medicine, Third Affiliated Hospital of Soochow University, Changzhou, China

**Keywords:** acute respiratory failure, vitamin D, mortality, prognosis, MIMIC-IV

## Abstract

**Background:**

Vitamin D plays a critical role in the regulation of multiple physiological pathways. Vitamin D deficiency may be a risk factor for life-threatening clinical conditions. Several studies have found that vitamin D supplementation in critically ill patients improves prognosis. The purpose of this study was to determine the association between vitamin D and the prognosis of patients with acute respiratory failure (ARF).

**Methods:**

In this retrospective cohort study, we collected clinical information of ARF patients from the Medical Information Mart for Intensive Care IV (MIMIC-IV) version 2.0 database. The outcome of this study was in-hospital mortality, intensive care unit (ICU) mortality. Patients were divided into the no-vitamin D and vitamin D groups according to whether they received supplementation or not. The correlation between vitamin D and outcome was examined using Kaplan–Meier (KM) survival curves, Cox proportional risk regression models and subgroup analyses. Propensity-score matching (PSM) was used to ensure the robustness of our findings.

**Results:**

The study finally included 7,994 patients with ARF, comprising 6,926 and 1,068 in the no-vitamin D and vitamin D groups, respectively. The Kaplan–Meier survival curve indicated a significant difference in survival probability between the two groups. After adjustment for a series of confounders, the multivariate Cox proportional hazards models showed that the hazard ratio (95% confidence interval) values for in-hospital and ICU mortality in the no-vitamin D group were 1.67 (1.45, 1.93) and 1.64 (1.36, 1.98), respectively. The results of propensity score-matched (PSM) analysis were consistent with the original population. In the subgroup analysis, Vitamin D supplementation was associated with lower in-hospital mortality in patients with higher clinical scores (SOFA score ≥ 8, OASIS ≥ 38).

**Conclusion:**

Our study concluded that Vitamin D supplementation may reduce in-hospital and ICU mortality in patients with ARF in the ICU. There may be a beneficial effect on in-hospital mortality in patients with higher clinical scores. Additional randomized controlled trials are needed to follow up to confirm the relationship between vitamin D supplementation and ARF.

## Introduction

Acute respiratory failure (ARF) is one of the common diseases in critical care wards and emergency departments. Severe acute respiratory failure manifests as respiratory distress with severe tachypnoea, labored breathing, and recruitment of accessory respiratory muscles. It can cause damage to heart, brain, kidney and other important organs due to hypoxia and carbon dioxide retention. There are many causes of acute respiratory failure, and the common causes are as follows. Pulmonary parenchymal lesions include severe pneumonia, emphysema, lung injury, pulmonary edema, etc., resulting in decreased lung volume and effective diffusion area, V/Q imbalance, and increased arteriovenous shunt (1). About 30% of patients admitted to the intensive care unit (ICU) are critical ARF patients. In addition, ARF patients in ICU have a higher mortality rate during hospitalization (2, 3).

Vitamin D is a lipid soluble vitamin with multiple physiological effects. Human endogenous vitamin D comes from the skin, and 7-dehydrocholesterol in subcutaneous tissue can synthesize vitamin D3 under the action of ultraviolet light. Exogenous vitamin D mainly comes from foods such as fish, eggs, milk, and animal organs (4). 25-OH-D3 is the main circulating form of vitamin D in the human body and is not regulated by calcium, phosphorus, and parathyroid hormones. It is the most commonly used indicator for detecting vitamin D content. 1,25- (OH) 2-D3 is the main active form of vitamin D and binds to vitamin D receptors to exert biological effects (5). Vitamin D receptors are widely expressed in myocardium, vascular smooth muscle, brain, liver, kidneys, bones, parathyroid glands, and various immune cells (6). Studies have shown that vitamin D is not only involved in calcium and phosphorus regulation, bone metabolism, but also in the regulation of the immune system, cell proliferation and differentiation, and cardiovascular system (7–10). Vitamin D deficiency has become a global public health problem, closely related to various diseases. Previous studies have shown that vitamin D deficiency is associated with osteoporosis, sarcopenia, cardiovascular disease, Alzheimer’s disease and diabetes (11–13). And a lot of studies showed that women or children over 6 years old with lower serum 25 (OH) D levels are more susceptible to pathogenic respiratory pathogens and vitamin D supplementation bring beneficial effects on acute respiratory infections (ARI) (14–16).

In consideration of evidence that hypovitaminosis D reduces innate cellular immunity, inhibits the cytokine storm and the impact on the prognosis of various diseases (17), we collected clinical information of ARF patients from the Medical Information Mart for Intensive Care IV (MIMIC-IV) version 2.0 database and assessed if vitamin D supplementation during ARF could further improve the prognosis of those patients.

## Methods

### Data source

We enrolled patients with ARF from the MIMIC-IV (Medical Information Mart for Intensive Care IV, version 2.0) database (18), which contains comprehensive data on 315,460 inpatients from 2008 to 2019. Informed consent was waived because the data were obtained from publicly available sources. we completed the Protecting Human Research Participants, an online course developed by the National Institutes of Health (author certification number: 49872601). The database was approved by the MIT Institutional Review Committee and Beth Israel Deaconess Medical Center. To protect patient privacy, all private information in the database repository has been deleted. The study was consistent with the Declaration of Helsinki compliant principles.

### Study population

In the MIMIC-IV database, we included patients diagnosed with acute respiratory failure at hospital admission. The codes of data extraction were International Classification of Diseases version 9 diagnosis codes (“51,851,” 51,881) and version 10 diagnosis codes (“J95821,” “J960,” “J9601,” “J9602”). The exclusion criteria were as follows: (1) aged<18 years; (2) patients with repeated hospital or ICU admissions; (3) Hospital or ICU stay less than 24 h. Patients were divided into the vitamin D and no vitamin D groups depending on whether or not they had received supplementation via oral routes.

### Data extraction and outcomes

We extracted the following variables from the MIMIC-IV version 2.0 database: demographic characteristics (age, gender, weight); vital signs, Laboratory parameters, and comorbidities. Vital signs included heart rate, systolic blood pressure (SBP), diastolic blood pressure (DBP), mean arterial pressure (MAP), respiratory rate, temperature, pulse oxygen saturation (SpO2) within 24 h of ICU admission. Laboratory parameters included hemoglobin, hematocrit, red cell distribution width (RDW), platelet count, anion gap, bicarbonate, chloride, bun, calcium, glucose, white blood cell (WBC) count, creatinine, prothrombin time (PT), International standard ratio (INR), serum sodium, and serum potassium within 24 h of ICU admission. Comorbidities included congestive heart failure (CHF), myocardial infarct, chronic pulmonary disease (CPD), rheumatic disease, peptic ulcer disease, renal failure, diabetes, malignant cancer, and liver disease. Sequential Organ Failure Assessment (SOFA), Oxford acute severity of illness score (OASIS), Acute Physiology Score III (APS III), ventilator use, vasopressor use, and renal replacement therapy (RRT) use were also included. The outcomes of this study were in-hospital and ICU mortality.

### Statistical analysis

Variables with >10% missing values were excluded, and the remaining missing data were estimated using mean imputations. The continuous variables in this study did not conform to a normal distribution, so they were expressed as median and interquartile (IQR) values, and the differences between the two groups were tested with the Mann–Whitney U test. For categorical variables, data were expressed as frequencies or percentages and analyzed using Chi-square or Fisher test.

Kaplan–Meier (KM) curves and log-rank tests were used to assess whether vitamin D administration affected patient survival. Multivariate Cox regression models were performed to estimate the association between Vitamin D supplementation and mortality in ARF patients. In Model 1, no covariates were adjusted. Model II was adjusted for the confounder’s age, and gender. Model III was adjusted for age, gender, hematocrit, hemoglobin, platelets, wbc, aniongap, bicarbote, bun, calcium, chlorid, creatinine, sodium, potassium, RDW, glucose, weight, heart rate, SBP, DBP, MAP, respiratory rate, temperature, SpO2, INR, PT, RRT, vasopressor_use, mechvent, apsiii, oasis, sofa, congestive heart failure, myocardial infarct, rheumatic disease, peptic ulcer disease, renal disease, diabetes, malignant cancer, and liver disease. To guarantee the robustness of the findings, we used propensity-score matching (PSM) to reduce the baseline differences between the two groups. Propensity score matching (PSM), at a ratio of 1:1, was performed using a caliper width of 0.02 of the SD of the logit of the propensity score. All data were analyzed were performed using R software (version 4.2.2) and SPSS version 23.0 (IBM Corp, Armonk, NY, United States). A *p* value < 0.05 was taken to indicate statistical significance.

## Results

### Baseline characteristics

**Figure 1:**
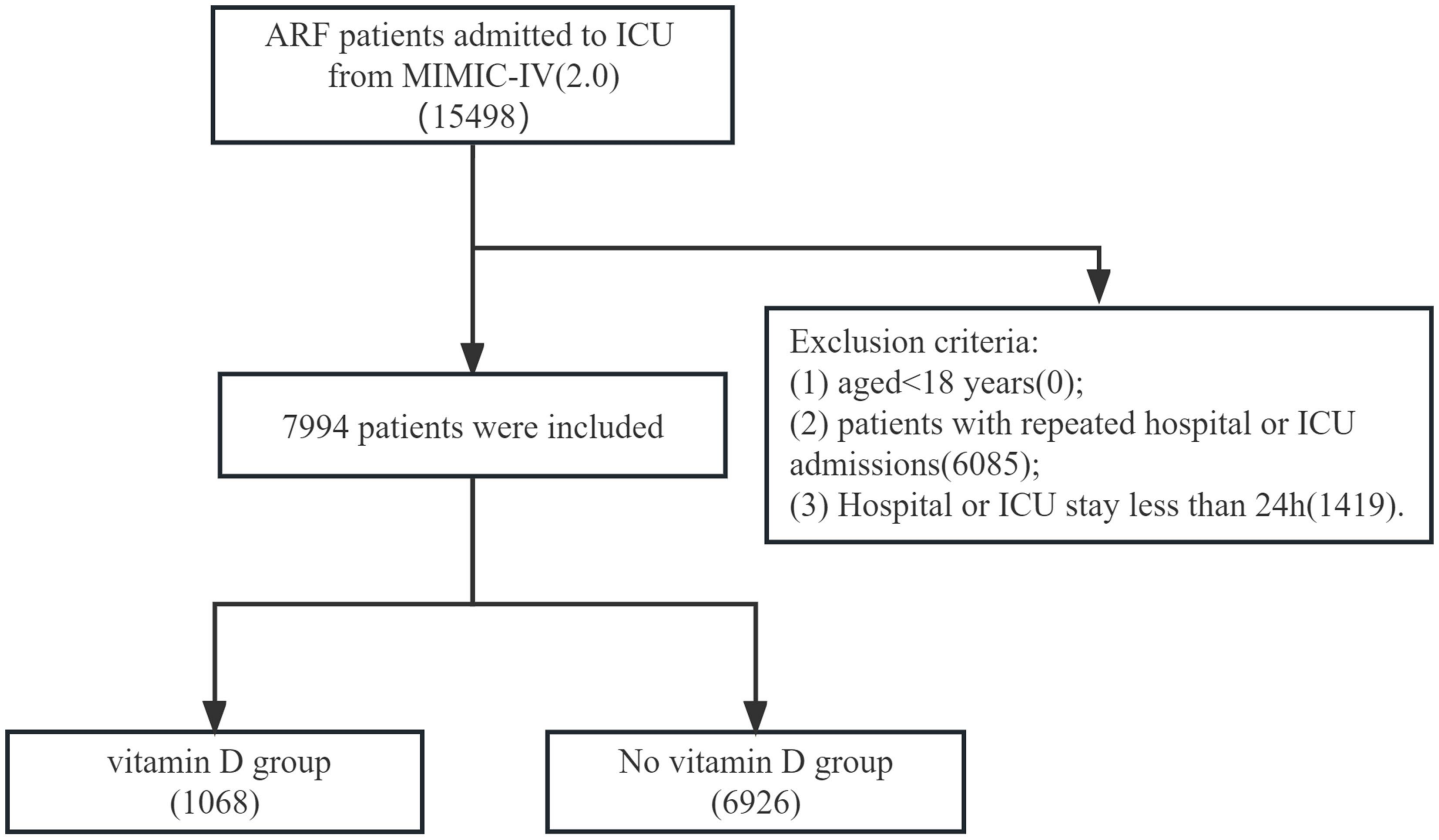
The flow chart of the included population.

A total of 7,994 patients meeting the selection criteria were included in this study, of which 6,926 in the no-vitamin D group and 1,068 in the vitamin D group (Figure 1). The first diagnosis of 7,994 patients are shown in Supplementary Table S1. Demographic characteristics, vital signs, laboratory indicators, and details of comorbidities at baseline are shown in Table 1. In the original population, the age of the vitamin D group was 72.16 (60.99, 81.89), which is lower than that of the no-vitamin D group 66.76 (54.88, 78.34). The SOFA Score and OASIS score in the no-vitamin D group were higher than those in the vitamin D group. There was also an increase in the use of ventilators and vasopressor in no-vitamin D patients. For patient outcomes, the no-vitamin D group had significantly higher in-hospital and ICU mortality rates.

**Table 1:** Baseline characteristics of the original population.

	Vitamin D	No vitamin D	*p*
*N*	1,068	6,926	
Age	72.16 (60.99, 81.89)	66.76 (54.88, 78.34)	<0.001
**Gender, *n***			
Female	576 (53.9%)	3,015 (43.5%)	<0.001
Male	492 (46.1%)	3,911 (56.5%)	
Weight (kg)	76.00 (63.45, 93.65)	80.00 (66.60, 97.19)	<0.001
**Vital signs**			
HR	88.31 (75.71, 99.29)	88.04 (77.67, 100.85)	0.23
SBP, mmHg	113.80 (105.43, 127.29)	113.71 (105.09, 125.95)	0.31
DBP, mmHg	60.92 (55.15, 67.96)	61.92 (55.46, 69.13)	0.02
MAP, mmHg	75.50 (69.60, 82.98)	76.44 (70.50, 83.96)	0.001
Respiratory rate (breaths/min)	20.40 (17.74, 23.59)	20.23 (17.62, 23.22)	0.25
Temperature	36.83 (36.59, 37.14)	36.94 (36.63, 37.33)	<0.001
SpO2, %	96.61 (95.01, 98.11)	97.07 (95.37, 98.58)	<0.001
**Laboratory parameters**			
RDW (%)	14.9 (13.92, 16.68)	14.61 (13.63, 16.04)	<0.001
Hemoglobin, g/dl	9.95 (8.50, 11.58)	10.75 (9.30, 12.40)	<0.001
Hematocrit, %	30.60 (26.30, 35.53)	32.90 (28.45, 37.75)	<0.001
Platelet, 10^9^ /L	200.00 (133.50, 266.0)	194.00 (135.00, 263.50)	0.23
WBC count, 10^9^ /L	11.60 (8.15, 15.93)	12.10 (8.85, 16.35)	0.005
Anion gap, mg/dl	15.00 (13.00, 17.50)	15.00 (13.00, 17.50)	0.9
Bicarbonate, mg/dl	22.50 (19.75, 26.00)	22.50 (19.50, 25.50)	<0.001
Bun, mg/dL	26.00 (17, 45)	23 (15, 38)	<0.001
Calcium, mg/dL	8.30 (7.83, 8.80)	8.20 (7.7, 8.7)	<0.001
Chloride, mmol/L	102.50 (98, 106)	104 (100, 108)	<0.001
Creatinine mg/dl	1.20 (0.8, 1.98)	1.1 (0.8, 1.8)	0.33
Glucose, mmol/L	136.50 (112, 175.75)	137.00 (114, 175)	0.11
Sodium, mg/dl	138 (135, 141.5)	139 (136, 141.5)	0.004
Potassium, mg/dl	4.20 (3.85, 4.65)	4.20 (3.85, 4.65)	0.72
INR	1.3 (1.1, 1.65)	1.3 (1.1, 1.65)	0.07
PT	14.2 (12.55, 17.95)	14.1 (12.5, 17.15)	0.17
**Severity of illness**			
SOFA score	7 (4, 10)	7 (5, 11)	<0.001
APS III score	58.00 (44, 79)	60 (43, 82)	0.23
OASIS	37 (31, 43)	39 (32, 45)	<0.001
**Comorbidities**			
Myocardial infarct, *n*	189 (17.7%)	1,260 (18.2%)	0.70
Congestive heart failure, *n*	473 (44.3%)	2,348 (33.9%)	<0.001
Rheumatic disease, *n*	73 (6.8%)	213 (3.1%)	<0.001
Chronic pulmory disease, *n*	404 (37.8%)	2,242 (32.4%)	<0.001
Peptic ulcer disease, *n*	31 (2.9%)	213 (3.1%)	0.76
Liver disease, *n*	188 (17.6%)	1,218 (17.6%)	0.99
Diabetes, *n*	388 (36.3%)	2020 (29.2%)	<0.001
Renal disease, *n*	323 (30.2%)	1,496 (21.6%)	<0.001
Malignant cancer, *n*	185 (17.3%)	1,099 (15.9%)	0.23
Ventilator use, *n*	529 (49.5%)	4,497 (64.9%)	<0.001
RRT use, *n*	75 (7%)	443 (6.4%)	0.44
Vasopressor use, *n*	142 (13.3%)	1,167 (16.8%)	0.003
**Mortality**			
In-hospital mortality, *n*	226 (21.2%)	1784 (25.8%)	0.001
ICU-mortality, *n*	126 (11.8%)	1,303 (18.8%)	<0.001

### Survival analysis and Cox proportional-hazards regression model

**Figure 2:**
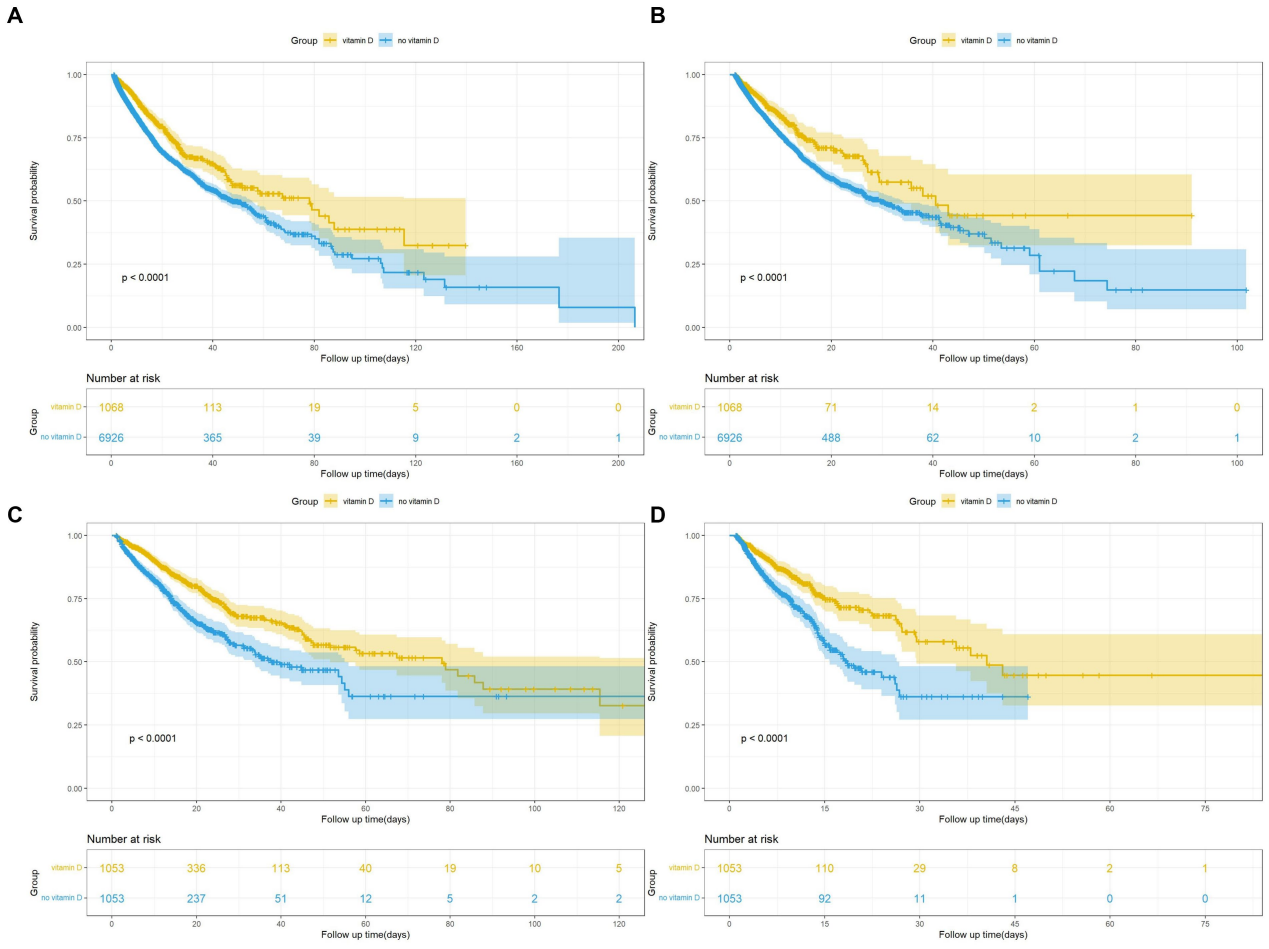
**(A)** Kaplan Meier curve of In-hospital mortality risk in two groups for the original population. **(B)** Kaplan Meier curve of ICU mortality risk in two groups for the original population. **(C)** Kaplan Meier curve of In-hospital mortality risk in two groups for the PSM population. **(D)** Kaplan Meier curve of ICU mortality risk in two groups for the PSM population.

**Figure 3:**
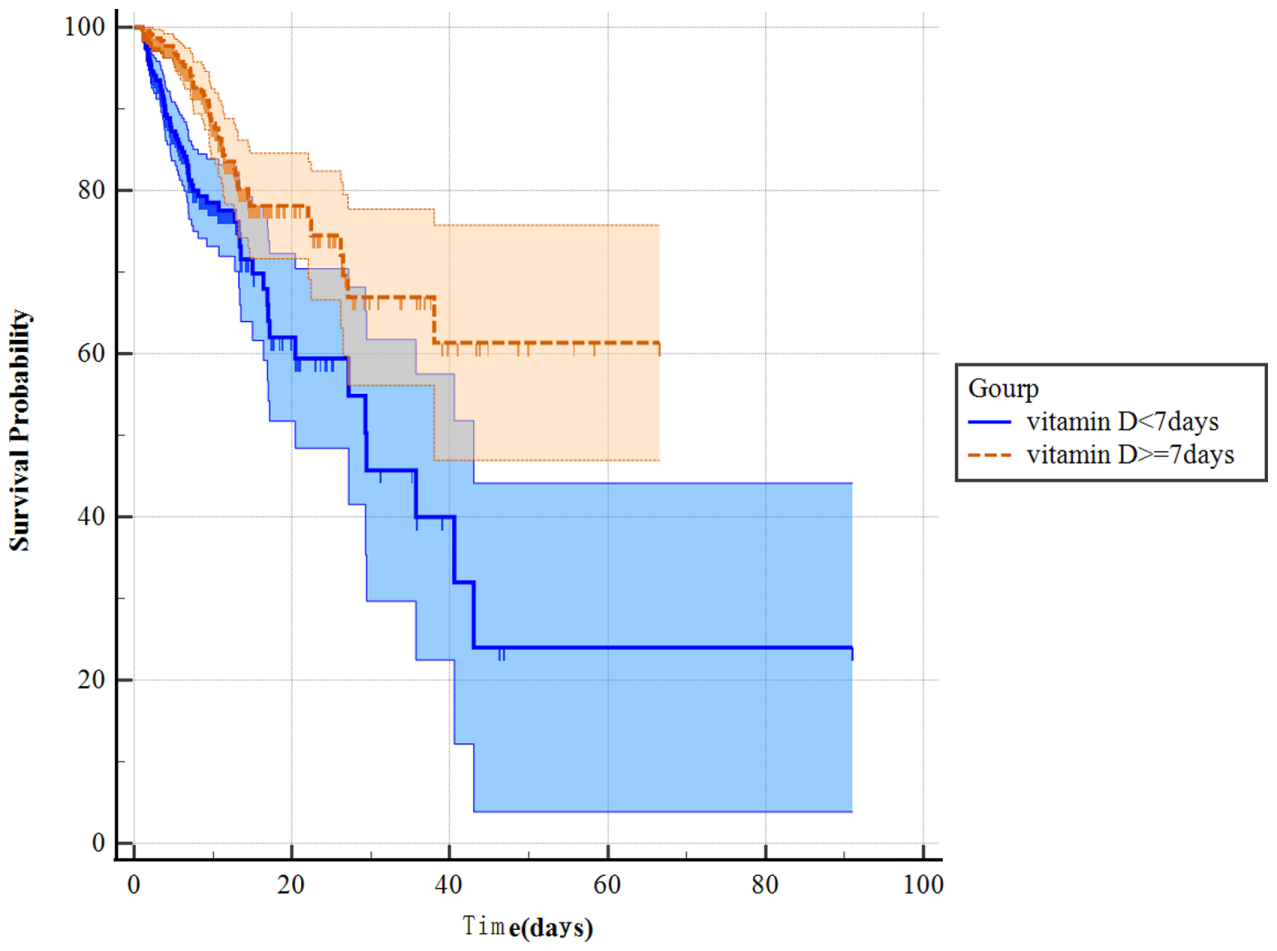
Kaplan Meier curve of ICU mortality risk in two groups for the original population.

The KM survival curves indicated that the survival probability differed significantly between the two groups (Figures 2A,B). Patients with ARF who received vitamin D had significantly higher survival odds in both the in-hospital and ICU (*p* < 0.001). Besides, we further analysis found that the survival probability in the group using vitamin D ≥ 7 days increased significantly compared to the group using vitamin D < 7 days (*p* < 0.001) (Figure 3). The Cox proportional-hazards model results are listed in Table 2. The raw model (model 1), which did not adjust for any variables, showed in-hospital and ICU mortality was significantly lower in the vitamin D group, compared to the no vitaminD group. Model 2, adjusted for sex and gender, still showed that vitamin D supplementation was associated with in-hospital and ICU mortality. In Model 3, after adjusting for confounding factors, the HR (95% confidence interval) values for in-hospital and ICU mortality in the no vitamin D group were 1.67 (1.45, 1.93) and 1.64 (1.36, 1.98), respectively, indicating that the in-hospital and ICU mortality risks were 1.67 and 1.64 times higher than those in the vitamin D group, respectively.

**Table 2:** Results of Cox proportional hazard models.

Outcomes	Model 1		Model 2		Model 3	
Original population	HR (95% CIs)	*p*-value	HR (95% CIs)	*p*-value	HR (95% CIs)	*p*-value
**In-hospital mortality**						
No-vitamin D	1.48 (1.29, 1.70)	<0.001	1.59 (1.38, 1.83)	<0.001	1.67 (1.45, 1.93)	<0.001
Vitamin D	1		1		1	
p for trend	<0.001		<0.001		<0.001	
**ICU-mortality**						
No-vitamin D	1.48 (1.23, 1.78)	<0.001	1.54 (1.28, 1.85)	<0.001	1.64 (1.36, 1.98)	<0.001
Vitamin D	1	<0.001	1	<0.001	1	<0.001
**PSM**						
**In-hospital mortality**						
No-vitamin D	1.69 (1.42, 2.02)	<0.001	1.65 (1.39, 1.97)	<0.001	1.67 (1.39, 2.00)	<0.001
Vitamin D	1		1		1	
p for trend		<0.001		<0.001		<0.001
**ICU-mortality**						
No-vitamin D	1.86 (1.48, 2.34)	<0.001	1.79 (1.42, 2.24)	<0.001	1.85 (1.46, 2.34)	<0.001
Vitamin D	1		1		1	

Model 1 covariates were adjusted for nothing.

Model 2 covariates were adjusted for age and gender.

Model 3 covariates were adjusted for age, gender, Hematocrit, Hemoglobin, Platelets, WBC, Anion gap, bicarbonate, Bun, Calcium, Chlorid, Creatinine, Sodium, Potassium, RDW, Glucose, Weight, Heart rate, SBP, DBP, MAP, Respiratory rate, Temperature, SpO2, INR, PT, RRT, Vasopressor use, Mechvent, APSIII, OASIS, SOFA, Congestive heart failure, Myocardial infarct, Chronic pulmory disease, Rheumatic disease, Peptic ulcer disease, Renal disease, Diabetes, Malignant cancer, and Liver disease.

### Propensity score matching

To reduce confounding bias, we performed PSM based on whether vitamin D was used. A total of 1,053 pairs were successfully matched. After matching, the baseline characteristics of patients in two groups were not significantly different (Table 3).

**Table 3:** Characteristics of the study population after propensity score matching.

	Vitamin D	No vitamin D	*p*-value
*N*	1,053	1,053	
Age (y)	71.79 (60.62, 81.58)	71.05 (60.03, 81.14)	0.54
**Gender, *n***			0.83
Female	563 (53.5%)	558 (53%)	
Male	490 (46.5%)	495 (47%)	
Weight (kg)	76.80 (63.5, 93)	77.40 (63.05, 92.15)	0.98
**Vital signs**			
HR	87.85 (75.71, 99.84)	87.84 (77.31, 99.42)	0.59
SBP, mmHg	114.29 (105.51, 127.27)	114.65 (105.6, 127.6)	0.78
DBP, mmHg	60.93 (55.15, 68.23)	62.04 (54.75, 69.6)	0.34
MAP, mmHg	75.48 (69.57, 83.04)	76.41 (69.91, 83.35)	0.30
Respiratory rate (breaths/min)	20.38 (17.75, 23.43)	20.41 (17.73, 23.34)	0.92
Temperature	36.84 (36.61, 37.14)	36.87 (36.62, 37.21)	0.14
SpO2, %	96.5 (94.96, 98.08)	96.64 (95, 98.24)	0.26
**Laboratory parameters**			
RDW (%)	14.85 (13.89, 16.47)	14.97 (13.91, 16.70)	0.18
Hemoglobin, g/dl	10.05 (8.60, 11.68)	10.15 (8.65, 11.70)	0.65
Hematocrit, %	31.10 (26.60, 35.78)	31.30 (27.05, 35.75)	0.65
Platelet, 10^9^ /L	199.50 (134.75, 266.50)	194.00 (134.0, 267.5)	0.71
WBC count, 10^9^ /L	11.45 (8.15, 15.58)	11.80 (8.53, 16.03)	0.10
Anion gap, mg/dl	15 (13, 17.5)	15 (12.5, 17.5)	0.72
Bicarbonate, mg/dl	23 (20, 26.5)	23 (20, 26)	0.56
Bun, mg/dL	24.5 (16.25, 43)	25.5 (16, 41.5)	0.95
Calcium, mg/dL	8.3 (7.9, 8.85)	8.3 (7.85, 8.8)	0.52
Chloride, mmol/L	102.50 (98, 106)	102.0 (98, 106.5)	0.76
Creatinine mg/dl	1.1 (0.8, 1.9)	1.1 (0.8, 1.8)	0.85
Glucose, mmol/L	135.50 (111.5, 172.5)	136.0 (113.5, 171.75)	0.62
Sodium, mg/dl	138.50 (135.5, 141.5)	138 (135.5, 141.0)	0.38
Potassium, mg/dl	4.2 (3.85, 4.65)	4.2 (3.85, 4.65)	0.76
INR	1.35 (1.15, 1.6)	1.3 (1.15, 1.6)	0.73
PT	14.7 (12.7, 17.25)	14.6 (12.8, 17.13)	0.83
**Severity of illness**			
SOFA score	6 (4, 10)	6 (4, 9)	0.47
APS III score	56 (43, 77.5)	56 (42, 76)	0.35
OASIS	37 (30, 43)	36 (30, 43)	0.46
**Comorbidities**			
Myocardial infarct, *n*	188 (17.9%)	181 (17.2%)	0.69
Congestive heart failure, *n*	462 (43.9%)	490 (46.5%)	0.22
Rheumatic disease, *n*	65 (6.2%)	67 (6.4%)	0.86
Chronic pulmory disease, *n*	396 (37.6%)	432 (41.0%)	0.11
Peptic ulcer disease, *n*	31 (2.9%)	26 (2.5%)	0.50
Liver disease, *n*	188 (17.9%)	190 (18.0%)	0.91
Diabetes, *n*	381 (36.2%)	372 (35.3%)	0.68
Renal disease, *n*	313 (29.7%)	309 (29.3%)	0.85
Malignant cancer, *n*	182 (17.2%)	181 (17.2%)	0.95
Ventilator use, *n*	528 (50.1%)	535 (5.1%)	0.76
RRT use, *n*	74 (7.0%)	61 (5.8%)	0.25
Vasopressor use, *n*	141 (13.4%)	130 (12.3%)	0.47
**Mortality**			
In-hospital mortality, *n*	220 (20.9%)	293 (27.8%)	<0.001
ICU-mortality, *n*	123 (11.7%)	198 (18.8%)	<0.001

The KM survival curves of the matched populations indicated a trend consistent with that for the original population (Figures 2C,D). As with the original population, we also applied multivariate Cox regression analyses to the matched populations. After the multivariate Cox regression, the HRs (95% CI) for in-hospital and ICU mortality in the no vitamin D group were 1.67 (1.39, 2.00) and 1.85 (1.46, 2.34), respectively, in the PSM population (Table 2).

### Subgroup analysis

We performed a subgroup analysis of the in-hospital and ICU mortality by using clinically significant scores and several complications (Table 4). A significant interaction was observed in clinically significant scores (SOFA score and oasis) on in-hospital mortality. For patients with higher clinical scores (SOFA score ≥ 8, OASIS ≥ 38), in-hospital mortality was higher in no vitamin D group. Therefore, The use of vitamin D has a significant protective effect on patients with higher clinical scores. No significant interactions were observed in other stratified population (p for interaction > 0.05).

**Table 4:** Subgroup analysis of relationship between groups and mortality.

		In-hospital mortality			ICU Mortality		
	*N*	HR (95%CI)	*p*-value	p-interaction	HR (95%CI)	*p* value	p-interaction
Gender				0.17			0.70
Male	4,403	1.61 (1.32, 1.97)	<0.001		1.63 (1.25, 2.14)	<0.001	
Female	3,591	1.76 (1.43, 2.17)	<0.001		1.74 (1.33, 2.28)	<0.001	
Age				0.57			0.42
≥60	5,328	1.56 (1.33, 1.83)	<0.001		1.49 (1.21, 1.83)	<0.001	
<60	2,666	1.58 (1.15, 2.17)	0.005		2.75 (1.72, 4.39)	<0.001	
SOFA score				**0.02**			0.10
<8	4,408	1.52 (1.22, 1.89)	<0.001		1.63 (1.18, 2.25)	0.003	
≥8	3,586	1.75 (1.45, 2.12)	<0.001		1.71 (1.35, 2.17)	<0.001	
APS III score				0.07			0.59
<63	4,462	1.65 (1.29, 2.12)	<0.001		1.82 (1.21, 2.74)	0.004	
≥63	3,532	1.69 (1.42, 2.02)	<0.001		1.62 (1.31, 2.01)	<0.001	
OASIS score				**0.008**			0.14
<38	3,826	1.62 (1.27, 2.05)	<0.001		1.76 (1.20, 2.58)	0.004	
≥38	4,168	1.75 (1.46, 2.10)	<0.001		1.69 (1.36, 2.10)	<0.001	
Congestive heart failure				0.23			0.52
No	5,173	1.84 (1.53, 2.23)	<0.001		1.82 (1.42, 2.33)	<0.001	
Yes	2,821	1.40 (1.12, 1.76)	0.003		1.46 (1.09, 1.97)	0.01	
Myocardial infarct				0.16			0.35
No	6,545	1.63 (1.39, 1.91)	<0.001		1.60 (1.30, 1.96)	<0.001	
Yes	1,449	1.78 (1.24, 2.55)	0.002		1.83 (1.14, 2.95)	0.01	
Chronic pulmonary disease				0.18			0.98
No	5,348	1.65 (1.39, 1.97)	<0.001		1.74 (1.38, 2.19)	<0.001	
Yes	2,646	1.68 (1.29, 2.18)	<0.001		1.49 (1.07, 2.07)	0.02	
Rheumatic disease				0.94			0.75
No	7,708	1.69 (1.46, 1.96)	<0.001		1.69 (1.39, 2.05)	<0.001	
Yes	286	1.52 (0.8, 2.80)	0.20		1.26 (0.53, 3.00)	0.60	
Peptic ulcer disease				0.33			0.25
No	7,750	1.67 (1.44, 1.93)	<0.001		1.62 (1.34, 1.95)	<0.001	
Yes	244	2.39 (0.81, 7.04)	0.12		2.46 (0.26, 23.43)	0.43	
Renal failure				0.40			0.21
No	6,175	1.72 (1.45, 2.04)	<0.001		1.66 (1.33, 2.07)	<0.001	
Yes	1819	1.58 (1.20, 2.08)	0.001		1.62 (1.12, 2.35)	0.01	
Liver disease				0.79			0.72
No	6,588	1.66 (1.40, 1.96)	<0.001		1.67 (1.34, 2.08)	<0.001	
Yes	1,406	1.84 (1.39, 2.44)	<0.001		1.91 (1.31, 2.77)	0.001	
Malignant cancer				0.71			0.45
No	6,710	1.75 (1.48, 2.07)	<0.001		1.78 (1.43, 2.21)	<0.001	
Yes	1,284	1.42 (1.06, 1.91)	0.02		1.33 (0.89, 1.98)	0.17	
Diabetes				0.84			0.77
No	5,586	1.71 (1.43, 2.03)	<0.001		1.76 (1.39, 2.23)	<0.001	
Yes	2,408	1.57 (1.22, 2.03)	0.001		1.46 (1.06, 2.02)	0.02	

HRs (95% CIs) were derived from Cox proportional hazards regression models. Covariates were adjusted as in model III (Table 2). The bold values indicate *p* < 0.05.

## Discussion

Respiratory failure is one of the most common reasons for hospitalization and intensive care unit admissions, and various etiologies can lead to it. According to the size of the hospital and the number of beds, patients with ARF can be managed in the ward or ICU. For patients who require ICU admission and invasive mechanical ventilation, the mortality rate before hospital discharge is up to 40% (3). Hence, recognizing and treating ARF in time lead to fewer complications, shorter ICU and hospital stay, and improved survival.

As we know, lung inflammation is the common reason leading to ARF, which is closely related to microbial infections. For example, coronavirus disease 2019 (Covid-19) can cause pneumonia, and severe patients may have respiratory failure or even death (19). In addition to anti-infective drugs, the immune status of the body and the inflammatory response in the body also affect the prognosis of the disease (20). Several studies suggested that vitamin D supplementation could potentially be effective either in treatment or prevention of Covid-19 (21, 22). The specific mechanism mainly includes the following three aspects. Firstly, vitamin D can induce various innate antimicrobial responses (23, 24). Secondly, vitamin D has anti-viral activities in respiratory viral infections (25). Furthermore, vitamin D could enhance cellular immunity by reducing the production of pro-inflammatory Th1 cytokines, such as tumor necrosis factor α and interferon γ (26), and increasing the expression of anti-inflammatory cytokines by macrophages (27). Besides, Olszowiec-Chlebna et al. also demonstrated vitamin D can modulate the innate immunity and inflammatory response to antigen challenge in airway cystic fibrosis patients (28). These reports showed the importance of vitamin D in infectious diseases.

A meta-analysis including more than 57,000 cases showed that vitamin D supplementation was related to the reduction of total mortality, and the mechanism may be related to the beneficial role of vitamin D in the cardiovascular system, the immune system, and the occurrence and development of tumors (29). A prospective study of patients with acute pancreatitis showed that serum 25-(OH)-D3 level was inversely related with the severity of acute pancreatitis as well as inflammatory markers. Moreover, they found that lower serum 25-(OH)-D3 level is a predictor for ICU admission and severe acute pancreatitis (30). Besides, a meta-analysis showed that vitamin D supplementation reduced the risk of cancer death by 16% (31). A study from multicenter, open-label, randomized controlled superiority trial showed that the early administration of high dose vitamin D3 to at-risk older patients with COVID-19 improved overall mortality at day 14 (32). However, the effect was no longer observed after 28 days. Another study about vitamin D supplementation in severe COVID-19 patients suggested that vitamin D supplementation had no benefit in patients with severe COVID-19 disease admitted to the ICU and in need of respiratory support (33). This result may be related to the small number of patients and many of patients had severe ARDS. Carpagnano et al. analyzed demographic, clinical and laboratory data of 42 patients with acute respiratory failure due to COVID-19 and the results showed that after 10 days of hospitalization, severe vitamin D deficiency patients had a 50% mortality probability, while those with vitamin D ≥ 10 ng/mL had a 5% mortality risk (34). However, they did not design to explore if vitamin D supplementation can improve prognosis.

The present study aimed to determine whether vitamin D supplementation may be beneficial in improving the prognosis of patients with acute respiratory failure in the Intensive Care Unit. In unadjusted analyses, we found the SOFA score and OASIS score in the no-vitamin D group were higher than those in the vitamin D group. And no-vitamin D supplementation group had significantly higher in-hospital and ICU mortality rates. In adjusted analyses, the baseline characteristics of patients in two groups were not significantly different, in-hospital and ICU mortality was still significantly lower in the vitamin D group.

The OASIS and SOFA score are classic tools for assessing the degree of illness in critically ill patients and the higher the score, the worse the prognosis of the disease (35, 36). Ricci et al. (37) analyzed 52 hospitalized patients affected with Covid-19 infection and with different degree of lung involvement and the results showed that patients with higher SOFA score displayed lower vitamin D level. Our subgroup analysis revealed that for patients with higher clinical scores (SOFA score ≥ 8, OASIS ≥ 38), in-hospital mortality was higher in no vitamin D group. Which means supplementation of vitamin D has a significant protective effect on patients with higher clinical scores. Our results provide favorable evidence for the application of vitamin D in patients with acute respiratory failure in the Intensive Care Unit.

Our study focused on the relationship between vitamin D supplementation and ARF and indicated vitamin D supplementation is beneficial in improving the prognosis of patients with acute respiratory failure in the Intensive Care Unit. However, our study has some limitations. Firstly, as a retrospective cohort study, there are still some unmeasured variables that may affect our results. Therefore, prospective studies are needed to confirm these results. Secondly, we cannot obtain the level of vitamin D in administration and the trends after treatment, which could reveal more information. Thirdly, although research has shown that vitamin D supplementation has important value in evaluating the prognosis of patients with acute respiratory failure, further research is needed to investigate its specific significance in different disease subgroups.

## Conclusion

Vitamin D supplementation can reduce in-hospital and ICU mortality in patients with ARF in the ICU. However, additional randomized controlled trials are needed to confirm the relationship between vitamin D supplementation and ARF.

## Data availability statement

The original contributions presented in the study are included in the article/Supplementary material, further inquiries can be directed to the corresponding author.

## Ethics statement

The studies involving humans were approved by Institutional Review Board of the Massachusetts Institute of Technology and Beth Israel Deaconess Medical Center. Written informed consent for participation was not required for this study in accordance with the National Legislation and the Institutional requirements.

## Author contributions

SH: Writing – original draft, Writing – review & editing. QH: Writing – original draft, Writing – review & editing. JX: Writing – review & editing. HL: Writing – review & editing, Data curation. RZ: Writing – review & editing. CL: Writing – review & editing, Writing – original draft.
