# Supplementary material for: Vitamin D supplementation is beneficial in improving the prognosis of patients with acute respiratory failure in the intensive care unit: a retrospective study based on the MIMIC-IV database
Source: Front Med (Lausanne). 2023 Nov 23;10:1271060. doi: 10.3389/fmed.2023.1271060 (PMC10708891; doi:10.3389/fmed.2023.1271060)
Supplement: Supplementary file 1 [file Table_1.DOCX]

**Table S1: The first diagnosis of the original population.**

|  | vitamin D | No vitamin D | P |
| --- | --- | --- | --- |
| N | 1068 | 6926 |  |
| Etiology |  |  | 0.23 |
| Sepsis | 321(30.1%) | 1985(28.7%) |  |
| Pneumonia | 170(15.9%) | 1087(15.7%) |  |
| heart failure | 157(14.7%) | 1048(15.1%) |  |
| Myocardial infarct | 106(9.9%) | 460(6.6%) |  |
| Acute kidney failure | 48(4.5%) | 352(5.1%) |  |
| Intracerebral hemorrhage | 64(6.0%) | 261(3.8%) |  |
| pulmonary embolism | 40(3.7%) | 188(2.7%) |  |
| COPD | 33(3.1%) | 642(9.3%) |  |
| pancreatitis | 32(3.0%) | 113(1.6%) |  |
| Malignant cancer | 28(2.6%) | 299(4.3%) |  |
| hematological disease | 34(3.2%) | 301(4.3%) |  |
| urinary tract diseases | 21(2.0%) | 86(1.2%) |  |
| Unidentified ARF | 14(1.3%) | 104(1.5%) |  |
